# The ubiquitin ligase RNF115 is required for the clearance of damaged lysosomes

**DOI:** 10.1002/1873-3468.70346

**Published:** 2026-04-24

**Authors:** Sae Nakanaga, Toshiki Takahashi, Akiko Kuma, Hiroyuki Kawahara

**Affiliations:** ^1^ Laboratory of Cell Biology and Biochemistry, Department of Biological Sciences Tokyo Metropolitan University Japan; ^2^ Graduate School of Medicine, Division of Health Sciences The University of Osaka Japan

**Keywords:** autophagy, BAG6, E3 ubiquitin ligase, lysophagy, lysosomal membrane damage, lysosome, RNF115

## Abstract

Lysosomes play a critical role in the quality control of cellular organelles. However, lysosomal membranes can be damaged under a variety of conditions, leading to the onset of various diseases. Damaged lysosomes are selectively cleared via a ubiquitin‐dependent mechanism, but the molecular mechanisms underlying this process have not been adequately elucidated. In this study, we found that RNF115 is a lysosomal damage‐responsive ubiquitin ligase that undergoes massive translocation from the cytosol to the p62/SQSTM1‐positive puncta around ruptured lysosomes. In accordance with the changes in its distribution, the depletion of RNF115 delayed the removal of Gal3 from damaged lysosomes during the restoration process following lysosomal damage. These observations suggest that RNF115 is responsible for the clearance of damaged lysosomes.

## Graphical abstract

Upon lysosomal rupture, an E3 ubiquitin ligase RNF115 translocates from the cytosol to the damaged lysosomal membrane. Moreover, RNF115 depletion impairs the clearance of damaged lysosomes, identifying it as a key regulator of lysosomal quality control. Future studies will uncover the direct mechanisms underlying this process, including recruitment of p62/SQSTM1 and Rab family small GTPases for lysophagy.

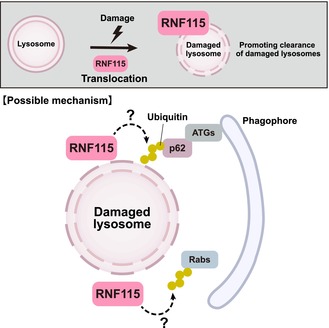

## Abbreviations

**ATG**, autophagy‐related

**EGFP**, enhanced green fluorescent protein

**Gal3**, galectin 3

**LAMP**, lysosomal‐associated membrane protein

**LLOMe**, L‐leucyl‐L‐leucine methyl ester

**RNF**, ring finger protein

**siRNA**, small interfering RNA

The lysosome is an acidic compartment that plays a critical role in the quality control of cellular proteins and organelles [1, 2, 3]. Despite the functional importance of lysosomes, lysosomal membranes can sometimes be damaged under a variety of physiological and pathological conditions. Lysosomal damage can result in the rupture of lysosomal membranes and the leakage of lysosomal contents [4, 5]. The release of lysosomal enzymes from the damaged lysosomes into the cytoplasm leads to oxidative stress, inflammation, and even cell death [4, 5, 6]. Indeed, the accumulation of damaged lysosomes can result in the development of various aging‐related diseases, including neurodegenerative diseases and hyperuricemic crystalline nephropathy [7]. Thus, the protective machinery against such lysosomal damage is critical for maintaining cellular homeostasis [8], and the mechanisms underlying the repair and elimination of damaged lysosomes have recently attracted considerable attention. However, the lysosomal damage response machinery has yet to be fully elucidated.

Lysosomal damage and the resulting membrane permeabilization can be experimentally induced by multiple means, including bacterial invasion, crystal and β‐amyloid formation, oxidative stress, and lysosomotropic chemicals [5, 6, 8, 9, 10, 11, 12]. A set of response pathways for lysosome rupture, collectively referred to as the lysosomal damage response, has been identified in eukaryotic cells [8, 13]. The lysosomal damage response involves the repair of the lysosomal membrane with endosomal sorting complexes required for transport (ESCRT), lysosomal regeneration by the transcription factor EB (TFEB)‐mediated translation cascade, and selective autophagy for irreparable lysosomes [2, 13, 14, 15, 16, 17, 18, 19].

Extensively damaged lysosomes are eliminated by autophagy through a ubiquitin‐ and autophagosome‐dependent mechanism, a process called lysophagy [13, 14, 15, 17, 20, 21]. In lysophagy, lysosomal membrane permeabilization leads to the formation of ubiquitin chains of exposed lysosomal membrane proteins and lipids [8, 15, 19, 22, 23], which triggers the recruitment of ubiquitin‐binding autophagy receptors such as p62/SQSTM1 [13, 23, 24, 25]. Several ubiquitin‐related enzymes have been identified in the lysosomal damage response. For example, substrate ubiquitination in damaged lysosomes is catalyzed by the E2 ubiquitin‐conjugating enzyme UBE2QL1 [22]. TRIM16 and neuron‐specific CUL1–SKP1–FBXO27 and FBXO2 are necessary for the formation of LC3‐positive ubiquitin puncta, which contribute to lysophagy [15, 26]. The CUL4A‐DDB1‐WDFY1 complex is also reported to ubiquitinate LAMP2 to promote the clearance of damaged lysosomal membranes [27]. Whereas knockdown of these ubiquitin ligases slows down the progression of lysophagy, it does not abolish it, suggesting that other ubiquitin ligases might be involved in the process of lysophagy. It is clear that further studies are needed to determine the machinery for the clearance of damaged lysosomes, which involves the contribution of the respective E3 ligases.

In the present study, we focused on this issue by investigating RNF115, also known as Rabring7 or BCA2. RNF115 is an RNF126‐related RING finger‐type E3 ubiquitin ligase [28, 29] that has been associated with intracellular membrane trafficking [29, 30, 31, 32, 33] and phagolysosomal trafficking responses [34, 35]. We found that RNF115 was recruited to the peripheral region of ruptured lysosomes following lysosomal damage induced by the lysosomotropic compound L‐leucyl‐L‐leucine methyl ester (LLOMe). RNF115 also colocalized with p62/SQSTM1‐positive structures upon LLOMe treatment. Notably, the depletion of RNF115 delayed the clearance of damaged lysosomes during the restoration process from LLOMe‐induced lysosomal damage. These observations collectively suggest that RNF115 is an essential ubiquitin ligase responsible for the clearance of damaged lysosomes.

## Materials and methods

### Plasmid construction

The mammalian expression vector encoding N‐terminal triple Flag‐tagged RNF115 (NM_014455.4) was generated in a previous study [29]. The siRNA‐insensitive RNF115 and the RNF115‐CS mutant constructs were generated using site‐directed mutagenesis PCR. For generation of the siRNA‐insensitive RNF115 construct, the following primers were used:

Sense, 5′‐TTTTTTCCACAGCAGTTGTATTGTGCCGTGGCTAG‐3′.

Antisense, 5′‐TGGTTACAAGGTAACTGCCGGACTTCCTCTTCAA‐3′.

For generation of the RNF115‐CS mutant, the following primers were used:

Sense, 5′‐AGAGGAAGTCCGGCAGTTACCTTGC‐3′.

Antisense, 5′‐TCAACTGTGTAATCTTCTTTGCTTACTGGACTCTC‐3′.

The mammalian expression vector for the N‐terminally triple T7‐tagged BAG6 N200 fragment was generated by PCR in a previous study [36].

All constructs were verified by Sanger sequencing prior to use.

### Mammalian cell culture and transfection

Authenticated human HeLa cells were purchased from RIKEN BRC (No# RCB0007, RRID:CVCL_0030). HeLa cells were cultured in Dulbecco's modified Eagle's medium (DMEM) (Wako, Osaka, Japan) containing 5% heat‐inactivated fetal bovine serum (FBS) (Nichirei, Tokyo, Japan) at 37 °C under a 5% CO_2_ atmosphere. HeLa cells stably expressing an enhanced green fluorescent protein (EGFP)‐tagged Gal3 were a generous gift from Prof. Kuma (The University of Osaka, Osaka, Japan) [27, 37] and were cultured in DMEM containing 10% heat‐inactivated FBS. All experiments were performed with mycoplasma‐free cells. For transient transfection, the expression vectors were transfected with Lipofectamine 2000 reagent (Invitrogen, Waltham, MA, USA), following the manufacturer's protocols. After 24‐h incubation, the cells were harvested and subjected to western blot or immunofluorescence analyses.

### RNA interference

*RNF115* and *ATG7* depletion in human cells was performed using the following sequences of the duplex siRNA (Sigma‐Aldrich, St. Louis, MO, USA).

5′‐CUUGCAAUCACUUCUUUCAtt‐3′ (*RNF115* #1).

5′‐GUUGAUAUGGGUUUAGAGUtt‐3′ (*RNF115* #2).

5′‐ GAGAUAUGGGAAUCCAUAAtt‐3′ (*ATG7*).

AllStars Negative Control siRNA (Qiagen, Venlo, Netherlands) was used as a general negative control in each experiment. Transfections of HeLa cells with duplex siRNA were performed using Lipofectamine™ RNAiMAX (Invitrogen, USA), following the manufacturer's protocol.

### Antibodies and reagent

Primary antibodies from mice were anti‐α‐tubulin monoclonal (T6199; Sigma‐Aldrich, USA), anti‐LAMP1 monoclonal (ab25630; Abcam, Cambridge, UK), anti‐vimentin monoclonal (sc‐6260; Santa Cruz Biotechnology, Dallas, TX, USA), anti‐p62/SQSTM1 monoclonal (M162‐3; MBL, Tokyo, Japan), and mouse anti‐Flag M2 monoclonal (F3165; Sigma‐Aldrich, USA). Primary antibodies generated by rabbits were anti‐RNF115 monoclonal (ab187642; Abcam, UK), anti‐ATG16L1 polyclonal (29445‐1‐AP; Proteintech, Rosemont, IL, USA), anti‐ATG7 monoclonal (sc‐376 212; Santa Cruz Biotechnology, USA), anti‐Flag polyclonal (F7425; Sigma‐Aldrich, USA), and anti‐T7 polyclonal (14441‐1‐AP; Proteintech, USA). Secondary fluorescent antibodies for western blotting were IRDye 800CW goat anti‐mouse/rabbit IgG and 680RD goat anti‐mouse/rabbit IgG (LI‐COR, Lincoln, NE, USA). The secondary fluorescent antibodies for immunocytochemistry were Alexa Fluor^R^488 goat anti‐mouse/rabbit IgG and Alexa Fluor^R^594 goat anti‐mouse/rabbit IgG (Invitrogen, USA).

LLOMe (HY‐129905A; MedChemExpress, Middlesex, NJ, USA) was used for the induction of lysosomal damage.

### Western blot analysis

Whole‐cell lysates and fractionated samples were subjected to SDS/PAGE and transferred onto polyvinylidene difluoride transfer membranes (IPFL00010 Immobilon‐FL; Merck Millipore, Burlington, MA, USA). The blots on the membranes were then visualized and quantified using a 2‐channel near‐infrared fluorescent imager (Odyssey DLx; LI‐COR, USA).

### Preparation of detergent‐insoluble fraction

HeLa cells cultured on 6‐well plates were washed twice with ice‐cold phosphate‐buffered saline (PBS) and then lysed on ice for 15 min with 200 μL of lysis buffer (PBS containing 1% Triton X‐100 and 20 μm MG‐132). After suspension by pipetting, 50 μL of cell lysate was collected as the total fraction. The remaining lysate was centrifuged at 20 000 *g* for 30 min at 4 °C to separate the detergent‐soluble fraction (supernatant) from the detergent‐insoluble fraction (pellet). After collecting the supernatant, the pellet was washed three times by resuspending it in lysis buffer and then centrifuging at 20 000 *g* for 30 min at 4 °C. The final pellet was resuspended in 200 μL of lysis buffer. Then, aliquots (50 μL each) of the total (whole), soluble, and insoluble fractions were subjected to western blot analysis.

### Immunofluorescence and microscopy

HeLa cells cultured on collagen‐coated micro cover glass (Matsunami, Osaka, Japan) were fixed with 4% paraformaldehyde (PFA) for 10 min, permeabilized with 0.1% Triton X‐100 for 3 min, and blocked with 3% calf serum solution in PBS for 30 min. For the immunofluorescence assay of endogenous RNF115 and GFP‐Gal3, cells were first fixed with 4% PFA for 15 min, followed by methanol fixation at 4°C for 5 min, and then blocked with 3% calf serum in PBS for 30 min. After that, cells were incubated with primary antibodies at 4 °C overnight. Subsequently, the samples were incubated with secondary antibody Alexa Fluor^R^488 goat anti‐mouse/rabbit IgG and Alexa Fluor^R^594 goat anti‐mouse/rabbit IgG for 1 h. Cover glasses were mounted on glass slides using Fluoromount mounting medium (Diagnostic BioSystems, Pleasanton, CA, USA). To observe the nucleus, cells were stained with 1 : 2500‐diluted Hoechst 33258 (H341; Dojindo, Kumamoto, Japan). Immunofluorescent images were obtained with an all‐in‐one fluorescence microscope (BZ‐X710; Keyence, Osaka, Japan) and an ECLIPSE Ti2‐E microscope equipped with an AX confocal microscope system operated by NIS‐Elements software (Nikon, Tokyo, Japan). Quantification of GFP‐Gal3 puncta and signal intensities for line profiling was analyzed using Fiji (ver. 2.14.0/1.54p).

### Gal3 dot‐count assay

HeLa cells stably expressing GFP‐Gal3 were cultured on collagen‐coated cover glasses. Cells were treated with 250 μm LLOMe for 1 h, washed twice with PBS, and incubated for the indicated time after washout. Cells were then fixed with 4% PFA for 15 min, and the cover glasses were mounted on glass slides using Fluoromount mounting medium. For nuclear staining, cells were incubated with 1 : 1000‐diluted Hoechst 33342 (#639; ImmunoChemistry Technologies, Davis, CA, USA). The number of puncta was analyzed using Fiji.

### Statistical analysis

Statistical analysis was performed using R (ver. 4.4.2) or Microsoft Excel from Microsoft Office 365. The statistical details of the experiments are stated in the legends of figures displaying the respective data, including the statistical tests used, the number of replicates, and the number of investigated cells, as well as the measures of precision. For two‐sample comparisons, two‐tailed Student's *t*‐test was used. For multiple comparisons, statistical significance was tested by Dunnett's multiple comparison test or Tukey's multiple comparison– test. A *P*‐value <0.05 was considered statistically significant (*P* < 0.05*, 0.01**, 0.001***).

## Results

### Lysosome rupture induces redistribution of RNF115 to the insoluble fraction

**Fig. 1:**
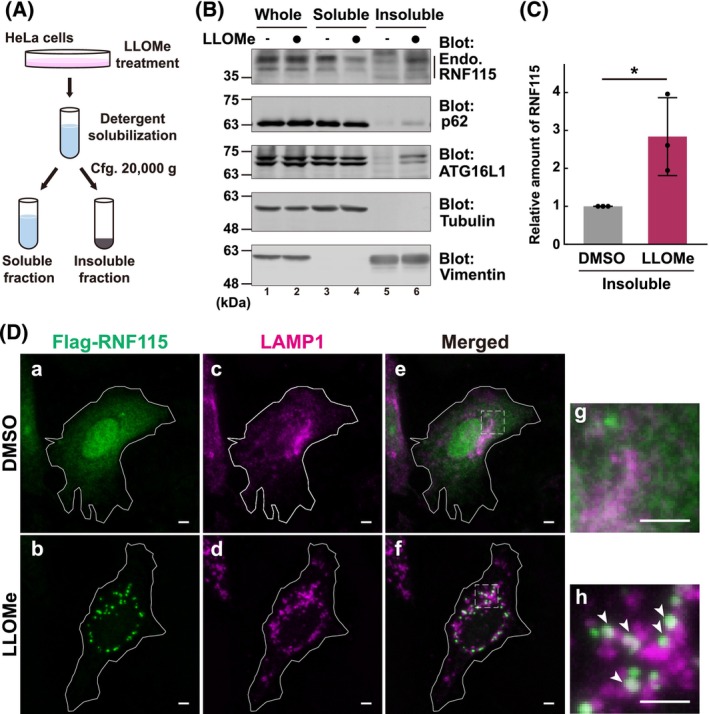
RNF115 translocated into the detergent‐insoluble fraction. (A) Schematic diagram of the detergent‐insoluble fractionation. (B) Western blotting analysis of detergent‐insoluble fractions derived from LLOMe‐treated or solvent (DMSO)‐treated HeLa cells. Representative data from three independent biological replicates are shown. HeLa cells were lysed with PBS containing 1% Triton X‐100 and fractionated by centrifugation into a supernatant (soluble fraction) and a pellet (insoluble fraction). (C) Quantification of RNF115 protein levels in the insoluble fraction from LLOMe‐treated cells or DMSO‐treated control cells. The signal intensities were normalized to those of vimentin (a marker for the detergent‐insoluble fraction). Data are presented as mean ± SD from three independent biological replicates. Statistical analysis was performed using a two‐tailed Student's *t*‐test (*P = 0.036). (D) RNF115 translocated to the foci close to a portion of the lysosomes following lysosomal damage, as visualized by fluorescence microscopy. HeLa cells expressing Flag‐tagged RNF115 were treated with 200 μm LLOMe or its solvent (DMSO) for 1 h and stained with anti‐Flag (shown in green) and anti‐LAMP1 (shown in magenta) antibodies. Typical images from a single experiment are shown. (a, b) Flag‐RNF115 stain. (c, d) LAMP1 stain. (e, f) Merged images. (a, c, e) DMSO‐treated cells. (b, d, f) LLOMe‐treated cells. (g, h) Enlarged views of the areas indicated by rectangles in (e) and (f), respectively. White lines indicate cell boundaries. White arrowheads in (h) indicate RNF115‐positive puncta adjacent to LAMP1‐positive vesicles. Scale bar, 5 μm.

In the fractionation analysis of HeLa cells (Fig. 1A), most of the endogenous RNF115 protein was detected in the soluble fraction, whereas only a trace amount of this protein was detected in the insoluble fractions (Fig. 1B, top panel, compare lanes 3 and 5). However, we found significant augmentation of endogenous RNF115 protein in the insoluble fractions derived from HeLa cells treated with LLOMe, a lysosome‐rupture reagent (Fig. 1B, top panel, compare lanes 5 and 6; see also Fig. 1C for quantification). RNF115 protein in the soluble fraction was slightly reduced by LLOMe treatment (Fig. 1B, top panel, compare lanes 3 and 4). These findings suggest that a portion of soluble RNF115 tends to be translocated to the insoluble fractions under lysosome‐rupture conditions.

The selective autophagy receptor p62/SQSTM1 showed a similar increase in the detergent‐resistant insoluble fractions with LLOMe treatment (Fig. 1B, second panel, compare lanes 5 and 6), as reported previously [38]. ATG16L1 is a constituent of the p62/SQSTM1 body, which can function as a nucleation center for the expanding phagophore to accelerate autophagosome maturation [39]. We observed increased incorporation of ATG16L1 into the RNF115‐ and p62/SQSTM1‐positive insoluble fractions after LLOMe treatment (Fig. 1B, third panel). These observations imply that RNF115 is a ubiquitin ligase responsive to lysosomal damage, which might be involved in the autophagic elimination of damaged lysosomes.

### RNF115 is recruited to the foci close to lysosomes following lysosomal damage

To directly visualize changes in RNF115 redistribution following lysosomal damage, we used immunocytochemistry to analyze the intracellular localization of Flag‐tagged RNF115. Consistent with the results obtained by cell fractionation analysis (Fig. 1A–C), Flag‐RNF115 was found to be a cytosolic protein uniformly distributed throughout the cytoplasm and the nucleus of non‐stimulated HeLa cells (Fig. 1D‐a). However, in LLOMe‐treated cells, cytosolic (and nuclear) signals of Flag‐RNF115 diminished (Fig. 1D‐b), in contrast to the case in non‐treated cells (Fig. 1D‐a). Notably, Flag‐RNF115 formed a number of cytoplasmic foci after treatment with LLOMe (Fig. 1D‐b). These RNF115‐positive puncta were colocalized with LAMP1 (Fig. 1D‐d,f), a general lysosomal marker [40]. Only some of the LAMP1‐positive vesicles were co‐stained with Flag‐RNF115 (Fig. 1D‐f,h, indicated by arrowheads), suggesting that a portion of the lysosome was labeled with RNF115.

**Fig. 2:**
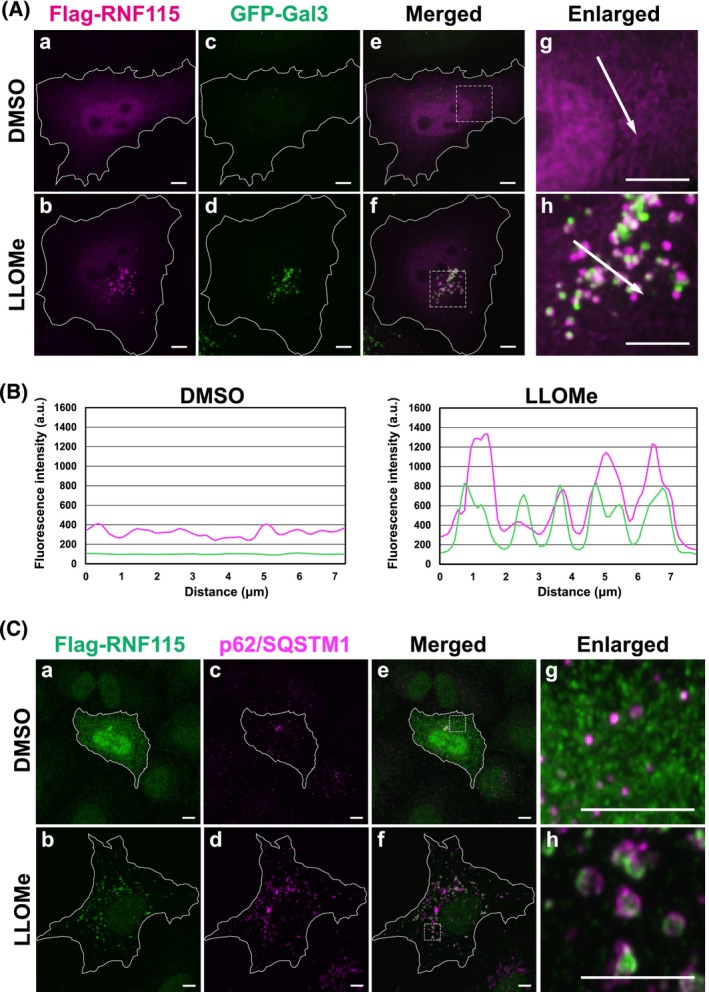
RNF115 translocated to the damaged lysosomes upon LLOMe treatment. (A, B) Confocal microscopic observations for HeLa cells stably expressing GFP‐Gal3 (green) and transiently expressing Flag‐RNF115 (magenta). Cells were treated with 250 μM LLOMe or its solvent (DMSO) for 1 h. Representative images from four independent experiments are shown. (a, b) Flag‐RNF115 stain. (c, d) GFP‐Gal3 signals. (e, f) Merged images. (g, h) Enlarged views of the areas indicated by rectangles in (e) and (f), respectively. (a, c, e, g) DMSO‐treated cells. (b, d, f, h) LLOMe‐treated cells. White lines indicate cell boundaries. Scale bar, 5 μm. See also Fig. S1. (B) Line profiles for Flag‐RNF115 signal (magenta) and GFP‐Gal3 signal (green) along the arrows indicated in the enlarged images in (g) and (h). (C) Confocal microscopic observations of HeLa cells stably expressing GFP‐Gal3 (green) and transiently expressing Flag‐RNF115 (magenta). Cells were treated with 250 μM LLOMe or its solvent (DMSO) for 1 h and stained with anti‐Flag and anti‐p62/SQSTM1 antibodies. Typical images from a single experiment are shown. (a, b) Flag‐RNF115 stain. (c, d) p62/SQSTM1 stain. (e, f) Merged images. (g, h) Enlarged views of the areas indicated by rectangles in (e) and (f), respectively. (a, c, e, g) DMSO‐treated cells. (b, d, f, h) LLOMe‐treated cells. White lines indicate cell boundaries. Scale bar, 5 μm.

To examine whether RNF115 is selectively associated with damaged lysosomes, we used HeLa cells stably expressing GFP‐fused Galectin3 (GFP‐Gal3) [27, 37]. Gal3 (β‐galactose‐binding lectin) is a damaged lysosomal marker that migrates into the lysosomal lumen from the cytosol upon lysosomal membrane permeabilization and binds to exposed luminal glycochains (Fig. 2A‐c,d). We found that most of the Flag‐tagged RNF115 immunosignals localized in the neighboring area of GFP‐Gal3 signals after LLOMe treatment (Fig. 2A‐b,d,f), although the RNF115 signal did not completely overlap with the Gal3‐positive puncta (Fig. 2A‐h). A line‐plot quantitative comparison of the signal distributions of RNF115 (magenta) and Gal3 (green) revealed a correlation between them (Fig. 2B), suggesting that RNF115 tends to translocate to the peripheral structure of the damaged lysosome. Similar to the case of Flag‐tagged RNF115, endogenous RNF115 was found to be localized in proximity to Gal3‐positive damaged lysosomes that appeared in LLOMe‐treated cells (Fig. S1). These observations suggest that part of RNF115 is recruited to the periphery of the damaged lysosome, forming the characteristic puncta.

To examine the potential involvement of RNF115‐positive puncta in lysophagy, we immunostained cells for Flag‐RNF115 and endogenous p62/SQSTM1, a selective autophagy receptor containing a UBA domain. We found that most RNF115 signal colocalized with p62/SQSTM1‐positive puncta after LLOMe treatment (Fig. 2C‐a–f). Interestingly, some of the RNF115 signals surrounding the p62/SQSTM1‐positive puncta appeared as vesicle‐like structures (Fig. 2C‐h). These findings suggest that RNF115 is associated with autophagy‐related proteins during the response to lysosomal damage, potentially contributing to the clearance of Gal3‐positive damaged lysosomes.

### RNF115 is required for the accelerated clearance of damaged lysosomes

**Fig. 3:**
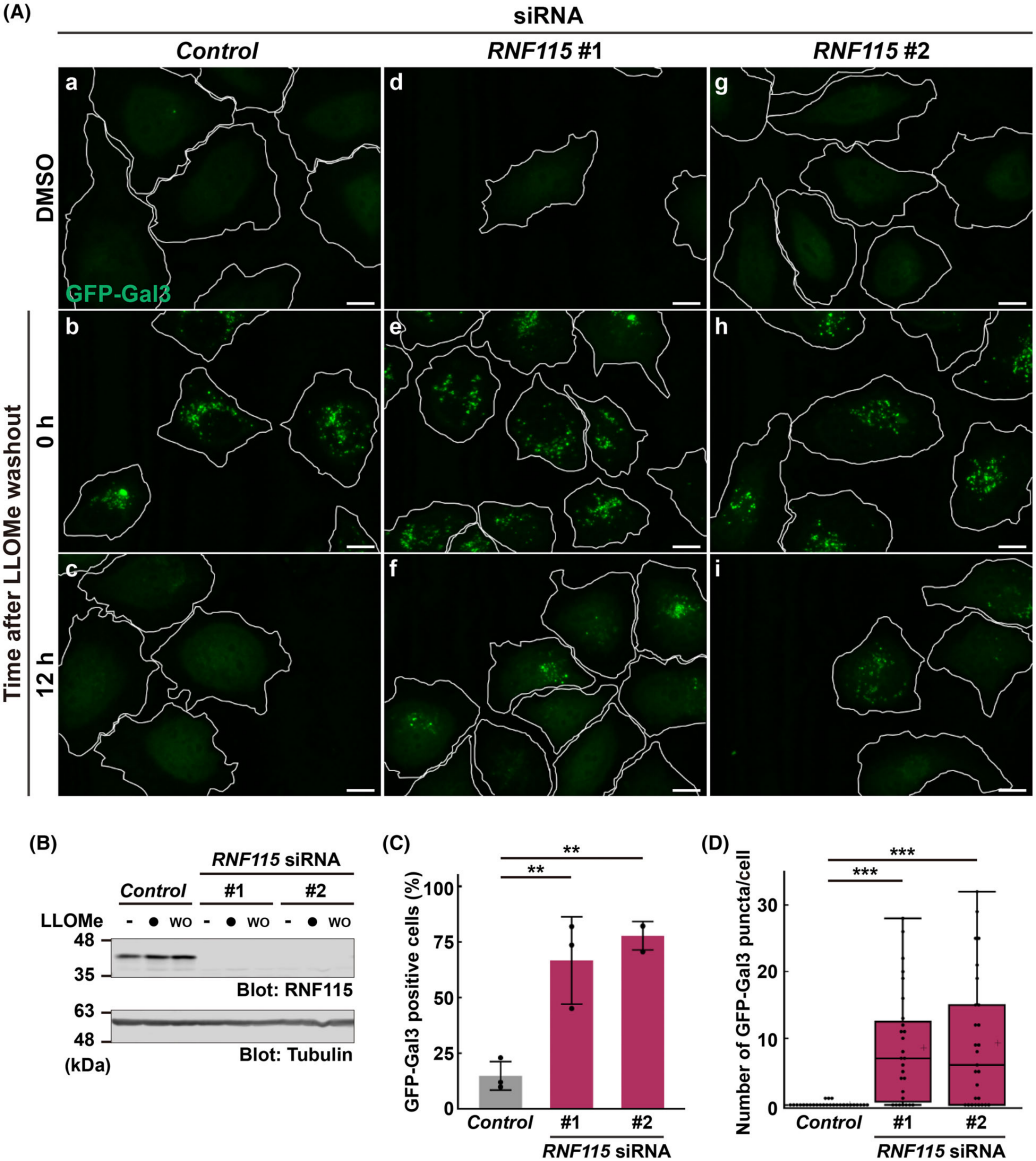
Depletion of RNF115 impairs the clearance of damaged lysosomes. (A) HeLa cells stably expressing GFP‐Gal3 (green) were transfected with *control* siRNA, or *RNF115* siRNAs #1 or #2 for 36 h and treated with 250 μm LLOMe or its solvent (DMSO) for 1 h. After LLOMe washout (this was defined as time zero), they were incubated for 12 h and subjected to fluorescence microscopy analysis for GFP‐Gal3. Representative images from three independent experiments are shown. (a–c) *Control* siRNA, (d–f) *RNF115* siRNA #1, and (g–i) *RNF115* siRNA #2. (a, d, g) DMSO‐treated cells. (b, e, h) LLOMe‐treated cells. (c, f, i) LLOMe‐washed out cells (12 h after washout). White lines indicate cell boundaries. Scale bar, 20 μm. See also Fig. S2. (B) Western blotting analysis confirming successful knockdown of RNF115 in HeLa cells. Representative data from three independent biological replicates are shown. WO, washout. (C) The percentage of GFP‐Gal3 puncta‐positive cells. Data are presented as mean ± SD from three independent experiments (*n* = 27 cells analyzed per condition in each experiment). Statistical analysis was performed using Dunnett's multiple comparison test (**P = 0.0040 for siRNA #1, **P = 0.0015 for siRNA #2). (D) The number of GFP‐Gal3 puncta per cell was automatically quantified. Data are presented as box‐and‐whisker plots, where the center line represents the median, the “+” symbol indicates the mean, the box indicates the interquartile range (IQR), and the whiskers extend to the minimum and maximum values. Data from a representative experiment are shown (*n* = 27 cells per condition). Similar results were obtained in three independent biological replicates. Statistical analysis was performed using Dunnett's multiple comparison test (***P = 0.0002 for siRNA #1, ***P < 0.0001 for siRNA #2).

Polyubiquitin modifications induced by several ubiquitin ligases are reported to act as a signal for the autophagic elimination of damaged lysosomes [15, 26, 27, 41]. Therefore, we investigated whether RNF115 is involved in the clearance of damaged lysosomes. The clearance kinetics of damaged lysosomes can be evaluated by using fluorescence microscopy to monitor the time‐dependent decrease in Gal3 puncta following LLOMe washout (Fig. 3A) [14, 27, 37, 42]. We quantified the number of GFP‐Gal3 puncta in RNF115‐depleted HeLa cells stably expressing GFP‐Gal3. After 1‐h LLOMe treatment, a significant number of GFP‐Gal3 puncta had formed (Fig. 3A‐b), unlike in the untreated cells (Fig. 3A‐a). These puncta almost completely disappeared within 12 h after LLOMe washout in the control cells (Fig. 3A‐c). The elimination of Gal3‐positive puncta after LLOMe washout is largely mediated by lysophagy, given that this process was blocked by the depletion of ATG7, an essential component of autophagy (Fig. S2), as previously suggested [14]. In contrast to control depletion, the Gal3 puncta remained in the cytoplasm even 12 h after LLOMe washout in RNF115‐depleted cells (Fig. 3A‐f,i, Fig. 3B), suggesting delayed clearance of damaged lysosomes. Statistical analysis of the three biologically independent replicates revealed a significant difference between RNF115‐knockdown cells and control‐knockdown cells at 12 h after LLOMe washout, both in terms of the percentage of GFP‐Gal3 puncta‐positive cells (Fig. 3C) and the number of GFP‐Gal3 puncta per cell (Fig. 3D). To avoid possible adventitious off‐target effects of each double‐stranded RNA, we designed two independent *RNF115* siRNA constructs targeting different sequences and provided evidence that these independent double‐stranded RNAs equally blocked the clearance of GFP‐Gal3 positive puncta after the removal of LLOMe (Fig. 3A, C, and D, siRNA #1 and #2). These observations suggest that endogenous RNF115 is essential for the clearance of damaged lysosomes.

### Excess expression of enzymatically inactive RNF115 blocks the clearance of damaged lysosomes

**Fig. 4:**
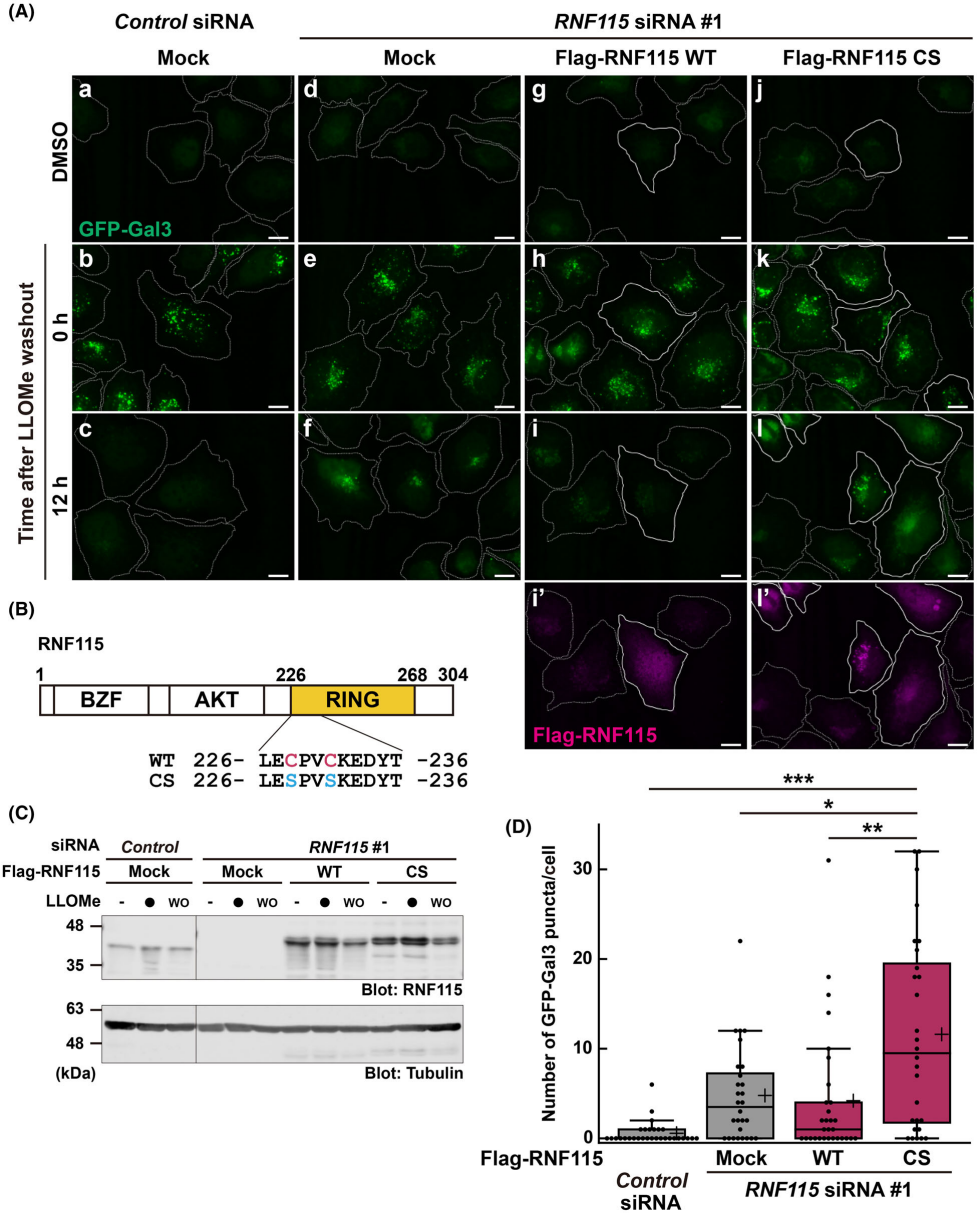
RING finger of RNF115 is essential for the clearance of damaged lysosomes. (A) HeLa cells stably expressing GFP‐Gal3 (green) were transfected with *control* siRNA or *RNF115* #1 siRNA for 12 h. Subsequently, the cells were transfected with siRNA‐insensitive forms of Flag‐RNF115 wild‐type (WT), CS mutant (CS), or empty (Mock) expression vectors. After 24 h, the cells were exposed to 250 μm LLOMe or its solvent (DMSO) for 1 h. Following LLOMe washout (this was defined as time zero), the cells were incubated for 12 h and subjected to fluorescence microscopy analysis for GFP‐Gal3. Typical images from a single experiment are shown. (a–c) *Control* siRNA + Mock expressions. (d–f) *RNF115* siRNA #1 + Mock expressions. (g–i, i') *RNF115* siRNA #1 + RNF115 WT expression. (j–l, l') *RNF115* siRNA #1 + RNF115 CS expressions. (a, d, g, j) DMSO‐treated cells. (b, e, h, k) LLOMe‐treated cells. (c, f, i, l, i', l') LLOMe‐washed out cells (12 h after washout). White lines and dashed lines indicate boundaries of Flag‐positive and Flag‐negative cells, respectively. (i', l') Flag‐RNF115 protein expressions were visualized by anti‐Flag immunostaining (shown in magenta). Scale bar, 20 μm. (B) Diagram of RNF115 domains. The amino acid sequences surrounding the cysteine‐to‐serine substitutions (C228S and C231S) within the RING finger domain, which inactivate the ubiquitin ligase activity, are shown. Amino acid numbers are denoted. (C) Western blotting analysis confirming depletions of endogenous RNF115 and the successful exogenous expression of Flag‐RNF115 proteins in HeLa cells. WO, washout. (D) The number of GFP‐Gal3 puncta per cell was automatically quantified. Data are presented as box‐and‐whisker plots, where the center line represents the median, the “+” symbol indicates the mean, the box indicates the interquartile range (IQR), and the whiskers extend to the minimum and maximum values. Data from a single experiment are shown (*n* = 27 cells per condition). Significant differences were observed by Tukey's multiple comparison test between the CS‐expressing cells and the following groups: *Contro*l siRNA + Mock (***P < 0.0001), *RNF115* siRNA #1 + Mock (*P = 0.0308), and *RNF115* siRNA #1 + WT (**P = 0.0012).

Overexpression of inactive RING finger ubiquitin ligases leads to stabilization of their substrates in a dominant‐negative fashion [43, 44]. Therefore, we attempted to overexpress the RING finger‐inactivated form of RNF115 mutant protein (in which Cys^228^ and Cys^231^ residues within the RING finger domain were substituted with serine in the siRNA‐insensitive mutant, designated as RNF115‐CS mutant, Fig. 4A, B, and C) for competitive inhibition of residual endogenous RNF115 protein in its pre‐depleted cells. Compared with the siRNA‐alone control, overexpression of the RNF115‐CS mutant led to a significant increase in GFP‐Gal3 puncta after LLOMe washout (Fig. 4A and D, *P < 0.05).

To examine whether the ubiquitin ligase activity of RNF115 is necessary for the clearance of damaged lysosomes, we compared the effects of exogenous expression of wild‐type (WT) RNF115 and the CS mutant on GFP‐Gal3 removal in RNF115‐depleted cells. We observed a reduction in the number of residual GFP‐Gal3 puncta in wild‐type RNF115‐expressed cells compared with RNF115‐knockdown cells (Fig. 4D), although the difference was not statistically significant. In contrast, cells expressing the RNF115‐CS mutant exhibited a significantly higher number of GFP‐Gal3 puncta compared to those expressing wild‐type RNF115 (Fig. 4D, **P < 0.01). These results suggest that the ubiquitin ligase activity of RNF115 influences the clearance kinetics of Gal3‐positive puncta after lysosomal damage.

## Discussion

In this paper, we found that RNF115 is a lysosomal damage‐responsive ubiquitin ligase that undergoes substantial changes in its subcellular localization after LLOMe treatment (Fig. 1). Although RNF115 is a soluble protein expressed uniformly throughout the cytoplasm, LLOMe treatment stimulates the massive translocation of RNF115 into the p62/SQSTM1‐positive cytoplasmic puncta around the damaged lysosomes (Fig. 2). The drastic changes in the distribution of RNF115 indicate a possible association with the clearance of damaged lysosomes. Strikingly, depletion of RNF115 significantly delayed the disappearance of the Gal3‐positive signal after washout of the lysosomal damage reagent (Fig. 3). Although Gal3 clearance is not specific to lysophagy, these observations suggest that RNF115 may play an essential role in the process of lysophagy.

**Fig. 5:**
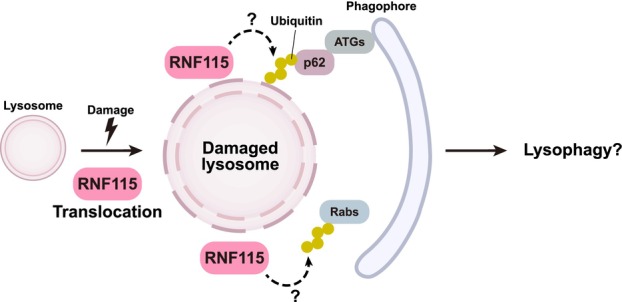
A hypothetical model of the RNF115‐mediated clearance of damaged lysosomes. Following lysosomal damage, RNF115 translocates to the cytoplasmic puncta around the damaged lysosomes. RNF115 might be an essential ubiquitin ligase that recruits the autophagy receptors (e.g., p62/SQSTM1), phagophore elongation factors (e.g., ATG16L1), and lysosomal membrane‐trafficking regulators (e.g., Rab family small GTPases) required to trigger lysophagy, although this has not yet been experimentally demonstrated.

In selective autophagy, ubiquitination is a known prerequisite for directing autophagic machinery to damaged organelles [23, 45, 46, 47, 48, 49, 50, 51]. Polyubiquitin chains connect damaged organelles with double membrane‐bound structures called autophagosomes via interaction with autophagy receptor proteins such as p62/SQSTM1 [23, 45, 52]. Previous studies have reported that SCF^FBXO27^ ubiquitinates exposed glycoproteins on damaged lysosomes to accelerate the recruitment of autophagic machinery and induce their clearance [15]. The CUL4‐based ubiquitin ligase complex was also identified as an E3 ligase involved in the ubiquitination of LAMP2 on damaged lysosomes [27]. In this context, the RNF115 may be involved in the ubiquitination of membrane proteins on the damaged lysosomes, as is the case in SCF^FBXO27^ and the CUL4‐based ubiquitin ligase complex (Fig. 5). However, our preliminary experiments failed to detect a significant decrease in anti‐ubiquitin immunosignal on damaged lysosomes in RNF115‐depleted cells. Although we still do not know how RNF115 supports lysosomal damage responses, RNF115 might regulate another layer of lysophagy progression, which is the membrane‐trafficking events necessary for the formation and fusion of phagosomes and phagolysosomes [53], given that RNF115 and its associated cofactors (e.g., BAG6) ubiquitinate several Rab family small GTPases (Fig. 5) [29, 33, 54, 55, 56, 57]. Notably, the BAG6 N‐terminal 200‐amino acid fragment (BAG6 N200), a substrate‐recognition domain of BAG6 [36, 58, 59], also changed its distribution after LLOMe treatment (Fig. S3). Because BAG6 is an RNF115‐associated chaperone‐like protein that is also responsible for the polyubiquitination of Rab family proteins [29, 55] and the N200 region of BAG6 is responsible for the recognition of ubiquitinated substrates for protein quality control [36, 59], RNF115 might regulate lysosome damage responses by collaborating with this protein quality control machinery. In relation to the above, RNF115 and BAG6 have also been reported to regulate endosomal sorting [29, 30, 32]. RNF115 and its related proteins promote autophagosome maturation, autophagosome‐lysosome fusion, and autophagic degradation under both nutrient‐enriched and stress conditions [34, 60], whereas Feng et al. recently reported that RNF115 suppresses autophagy progression [61]. Obviously, it is necessary to clarify the reason for this discrepancy. An important issue that remains to be elucidated is the identification of RNF115 substrates after lysosomal damage. Future studies should aim to elucidate the critical function and substrates of RNF115 in the clearance of damaged lysosomes.

## Author contributions

SN; conceived and designed the experiments, performed all the experiments, analyzed data, performed the statistical analysis, wrote the manuscript. TT; conceived and designed the experiments, analyzed data. AK; conceived and designed the experiments. HK; conceived and designed the experiments, analyzed data, wrote the manuscript, and supervised the study.

## Supporting information


**Fig. S1.** Related to Fig. 2A, endogenous RNF115 localized in proximity to Gal3‐positive damaged lysosomes upon LLOMe treatment. HeLa cells stably expressing GFP‐Gal3 (green) were treated with 250 μm LLOMe or its solvent (DMSO) for 1 h and then stained with anti‐RNF115 antibody (magenta). Typical images from a single experiment are shown. (a, b) Endogenous RNF115 stain. (c, d) GFP‐Gal3 signals. (e, f) Merged images. (a, c, e, g) DMSO‐treated cells. (b, d, f, h) LLOMe‐treated cells. White arrowheads in (f) indicate endogenous RNF115 signal in proximity to GFP‐Gal3 signal. (g, h) Overview images of the DMSO‐ or LLOMe‐treated cells. White lines indicate cell boundaries, and dashed rectangles indicate the enlarged areas shown in (a–f). Scale bar, 5 μm.

**Fig. S2.** Related to Fig. 3, ATG7 is required for the elimination of damaged lysosomes. (A) HeLa cells stably expressing GFP‐Gal3 (green) were transfected with *control* siRNA or *ATG7* siRNA for 36 h and treated with 250 μm LLOMe or its solvent (DMSO) for 1 h. After LLOMe washout (this was defined as time zero), the cells were incubated for 12 h and subjected to fluorescence microscopy analysis for GFP‐Gal3. Representative images from three independent experiments are shown. (a–c) *Control* siRNA and (d–f) *ATG7* siRNA. (a, d) DMSO‐treated cells. (b, e) LLOMe‐treated cells. (c, f) LLOMe‐washed out cells (12 h after washout). White lines indicate cell boundaries. Scale bar, 20 μm. (B) Western blotting analysis confirming *ATG7* knockdown in HeLa cells. Representative data from three independent biological replicates are shown. WO, washout. (C) The percentage of GFP‐Gal3 puncta‐positive cells. Data are presented as mean ± SD from three independent experiments (*n* = 27 cells analyzed per condition in each experiment). Statistical analysis was performed using a two‐tailed Student's *t*‐test (*P = 0.0109). (D) The number of GFP‐Gal3 puncta per cell was automatically quantified. Data are presented as box‐and‐whisker plots, where the center line represents the median, the “+” symbol indicates the mean, the box indicates the interquartile range (IQR), and the whiskers extend to the minimum and maximum values. Data from a representative experiment are shown (*n* = 27 cells per condition). Similar results were obtained in three independent biological replicates. Statistical analysis was performed using a two tailed Student's *t*‐test (***P = 0.0002).

**Fig. S3.** The substrate‐recognition domain of BAG6 (BAG6 N200) is recruited to lysosomes upon LLOMe treatment. Confocal microscopy observations of T7‐BAG6 N200 (T7‐tagged N‐terminal region of BAG6) in HeLa cells. BAG6 N200 is a fragment required for both substrate recognition and RNF115 association [28, 36, 59]. Cells were treated with 250 μm LLOMe or its solvent (DMSO) for 1 h and then stained with anti‐T7 tag (green) and anti‐LAMP1(magenta) antibodies. Typical images from a single experiment are shown. (a, b) T7‐BAG6 N200 stain. (c, d) LAMP1 stain. (e, f) Merged images. (a, c, e) DMSO‐treated cells. (b, d, f) LLOMe‐treated cells. (a'–f') Enlarged views of the areas indicated by rectangles in (a–f), respectively. White lines indicate cell boundaries. Note that all images presented in this figure were acquired in an identical set of experiments and that the exposure times of the respective immunostainings in this figure were the same. T7‐tagged BAG6 N200 was found to translocate to the foci close to the lysosome marker LAMP1 following LLOMe‐induced lysosomal damage. Some of the BAG6 N200 signals surrounding the LAMP1‐positive puncta appeared as vesicle‐like structures (b', f'). Scale bar, 5 μm.

## Data Availability

Unique reagents and data generated in this study are available from the corresponding author.
