# Supplementary material for: The ubiquitin ligase RNF115 is required for the clearance of damaged lysosomes
Source: FEBS Lett. 2026 Apr 24;600(13):1872–84. doi: 10.1002/1873-3468.70346 (PMC13358411; doi:10.1002/1873-3468.70346)
Supplement: Supplementary file 1 — Fig. S1. Related to Fig. 2A, endogenous RNF115 localized in proximity to Gal3‐positive damaged lysosomes upon LLOMe treatment. HeLa cells stably expressing GFP‐Gal3 (green) were treated with 250 μm LLOMe or its solvent (DMSO) for 1 h and then stained with anti‐RNF115 antibody (magenta). Typical images from a single experiment are shown. (a, b) Endogenous RNF115 stain. (c, d) GFP‐Gal3 signals. (e, f) Merged images. (a, c, e, g) DMSO‐treated cells. (b, d, f, h) LLOMe‐treated cells. White arrowheads in (f) indicate endogenous RNF115 signal in proximity to GFP‐Gal3 signal. (g, h) Overview images of the DMSO‐ or LLOMe‐treated cells. White lines indicate cell boundaries, and dashed rectangles indicate the enlarged areas shown in (a–f). Scale bar, 5 μm. [file FEB2-600-1872-s002.pdf]

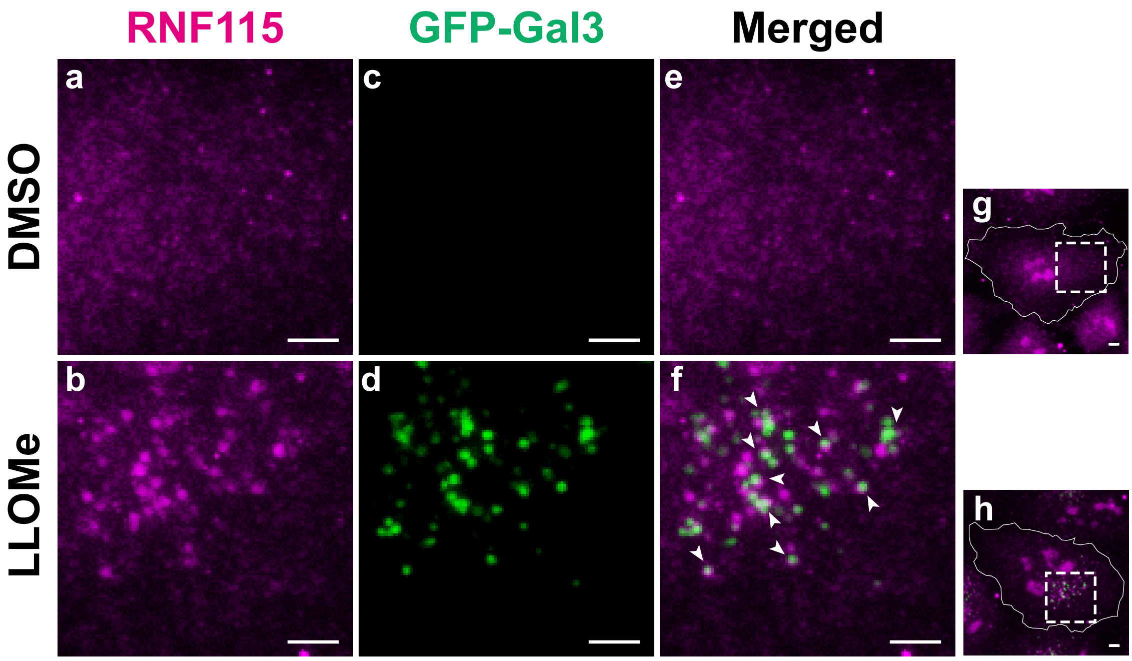

Fig. S1. Related to Fig. 2A, endogenous RNF115 localized in proximity to Gal3-positive damaged lysosomes upon LLOMe treatment.
HeLa cells stably expressing GFP-Gal3 (green) were treated with 250 µM LLOMe or its solvent (DMSO) for 1 h and then stained with anti-RNF115 antibody (magenta). Typical images from a single experiment are shown. **(a, b)** Endogenous RNF115 stain. **(c, d)** GFP-Gal3 signals. **(e, f)** Merged images. (**a, c, e, g**) DMSO-treated cells. (**b, d, f, h**) LLOMe-treated cells. White arrowheads in (f) indicate endogenous RNF115 signal in proximity to GFP-Gal3 signal. (**g, h**) White lines indicate cell boundaries, and dashed rectangles indicate the enlarged areas shown in (a–f). Scale bar, 5 µm.
