# Supplementary material for: The ubiquitin ligase RNF115 is required for the clearance of damaged lysosomes
Source: FEBS Lett. 2026 Apr 24;600(13):1872–84. doi: 10.1002/1873-3468.70346 (PMC13358411; doi:10.1002/1873-3468.70346)
Supplement: Supplementary file 2 — Fig. S2. Related to Fig. 3, ATG7 is required for the elimination of damaged lysosomes. (A) HeLa cells stably expressing GFP‐Gal3 (green) were transfected with control siRNA or ATG7 siRNA for 36 h and treated with 250 μm LLOMe or its solvent (DMSO) for 1 h. After LLOMe washout (this was defined as time zero), the cells were incubated for 12 h and subjected to fluorescence microscopy analysis for GFP‐Gal3. Representative images from three independent experiments are shown. (a–c) Control siRNA and (d–f) ATG7 siRNA. (a, d) DMSO‐treated cells. (b, e) LLOMe‐treated cells. (c, f) LLOMe‐washed out cells (12 h after washout). White lines indicate cell boundaries. Scale bar, 20 μm. (B) Western blotting analysis confirming ATG7 knockdown in HeLa cells. Representative data from three independent biological replicates are shown. WO, washout. (C) The percentage of GFP‐Gal3 puncta‐positive cells. Data are presented as mean ± SD from three independent experiments (n = 27 cells analyzed per condition in each experiment). Statistical analysis was performed using a two‐tailed Student's t‐test (*P = 0.0109). (D) The number of GFP‐Gal3 puncta per cell was automatically quantified. Data are presented as box‐and‐whisker plots, where the center line represents the median, the “+” symbol indicates the mean, the box indicates the interquartile range (IQR), and the whiskers extend to the minimum and maximum values. Data from a representative experiment are shown (n = 27 cells per condition). Similar results were obtained in three independent biological replicates. Statistical analysis was performed using a two tailed Student's t‐test (***P = 0.0002). [file FEB2-600-1872-s003.pdf]

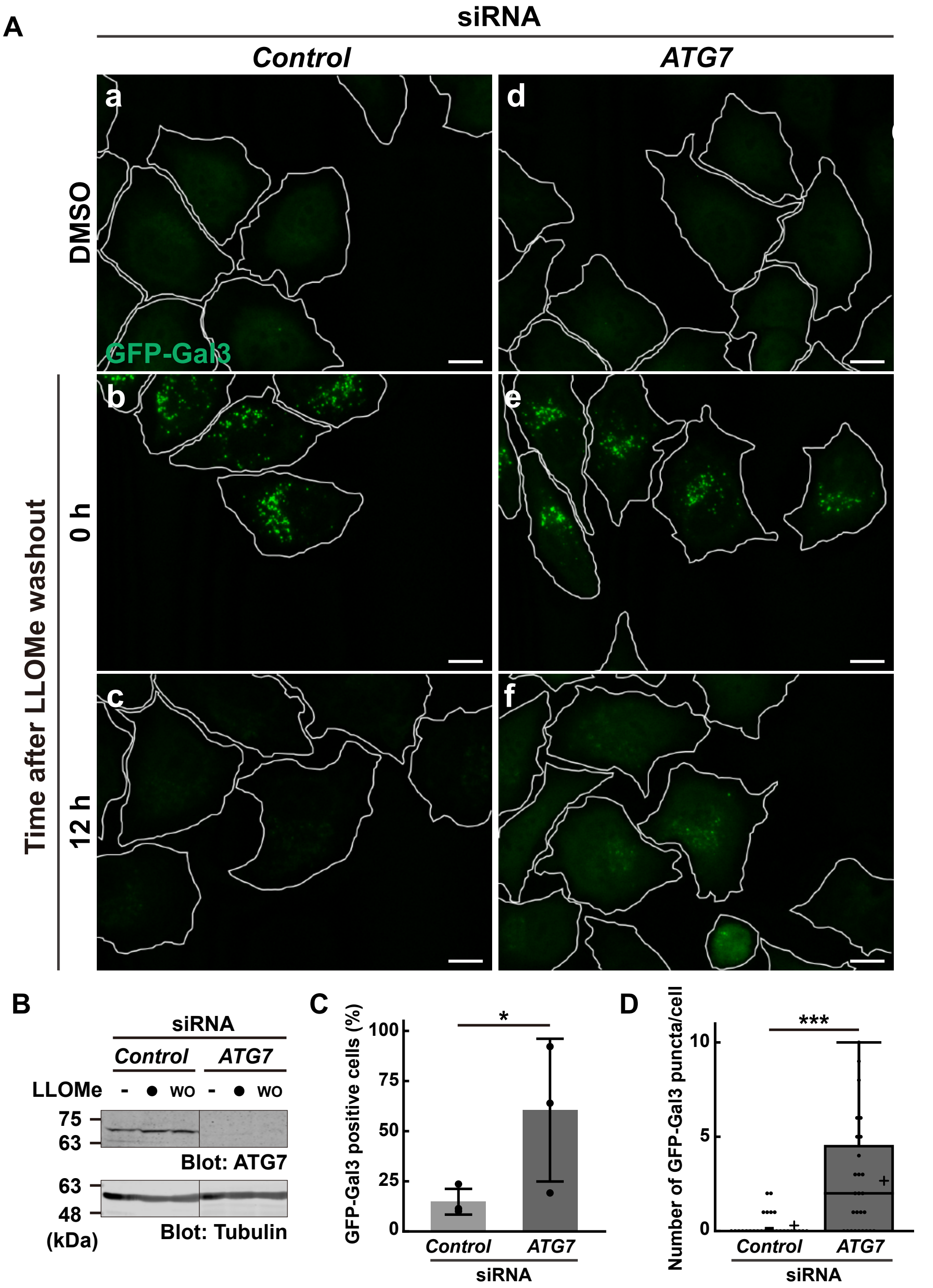


**Fig. S2. Related to Fig. 3, ATG7 is required for the elimination of damaged lysosomes.**
**(A)** HeLa cells stably expressing GFP-Gal3 (green) were transfected with *control* siRNA or *ATG7* siRNA for 36 h and treated with 250 µM LLOMe or its solvent (DMSO) for 1 h. After LLOMe washout (this was defined as time zero), the cells were incubated for 12 h and subjected to fluorescence microscopy analysis for GFP-Gal3. Representative images from three independent experiments are shown. **(a–c)** *Control* siRNA and **(d–f)** *ATG7* siRNA. (**a, d**) DMSO-treated cells. (**b, e**) LLOMe-treated cells. (**c, f**) LLOMe-washed out cells (12 h after washout). White lines indicate cell boundaries. Scale bar, 20 µm.
**(B)** Western blotting analysis confirming *ATG7* knockdown in HeLa cells. Representative data from three independent biological replicates are shown. WO, washout.
**(C)** The percentage of GFP-Gal3 puncta-positive cells. Data are presented as mean ± SD from three independent experiments ($n$ = 27 cells analyzed per condition in each experiment). Statistical analysis was performed using a two-tailed Student's $t$-test (*$p$ = 0.0109).
**(D)** The number of GFP-Gal3 puncta per cell was automatically quantified. Data are presented as box-and-whisker plots, where the center line represents the median, the "+" symbol indicates the mean, the box indicates the interquartile range (IQR), and the whiskers extend to the minimum and maximum values. Data from a representative experiment are shown ($n$ = 27 cells per condition). Similar results were obtained in three independent biological replicates. Statistical analysis was performed using Student's $t$-test (***$p$ = 0.0002).
