# Supplementary material for: The ubiquitin ligase RNF115 is required for the clearance of damaged lysosomes
Source: FEBS Lett. 2026 Apr 24;600(13):1872–84. doi: 10.1002/1873-3468.70346 (PMC13358411; doi:10.1002/1873-3468.70346)
Supplement: Supplementary file 3 — Fig. S3. The substrate‐recognition domain of BAG6 (BAG6 N200) is recruited to lysosomes upon LLOMe treatment. Confocal microscopy observations of T7‐BAG6 N200 (T7‐tagged N‐terminal region of BAG6) in HeLa cells. BAG6 N200 is a fragment required for both substrate recognition and RNF115 association [28, 36, 59]. Cells were treated with 250 μm LLOMe or its solvent (DMSO) for 1 h and then stained with anti‐T7 tag (green) and anti‐LAMP1(magenta) antibodies. Typical images from a single experiment are shown. (a, b) T7‐BAG6 N200 stain. (c, d) LAMP1 stain. (e, f) Merged images. (a, c, e) DMSO‐treated cells. (b, d, f) LLOMe‐treated cells. (a'–f') Enlarged views of the areas indicated by rectangles in (a–f), respectively. White lines indicate cell boundaries. Note that all images presented in this figure were acquired in an identical set of experiments and that the exposure times of the respective immunostainings in this figure were the same. T7‐tagged BAG6 N200 was found to translocate to the foci close to the lysosome marker LAMP1 following LLOMe‐induced lysosomal damage. Some of the BAG6 N200 signals surrounding the LAMP1‐positive puncta appeared as vesicle‐like structures (b', f'). Scale bar, 5 μm. [file FEB2-600-1872-s001.pdf]

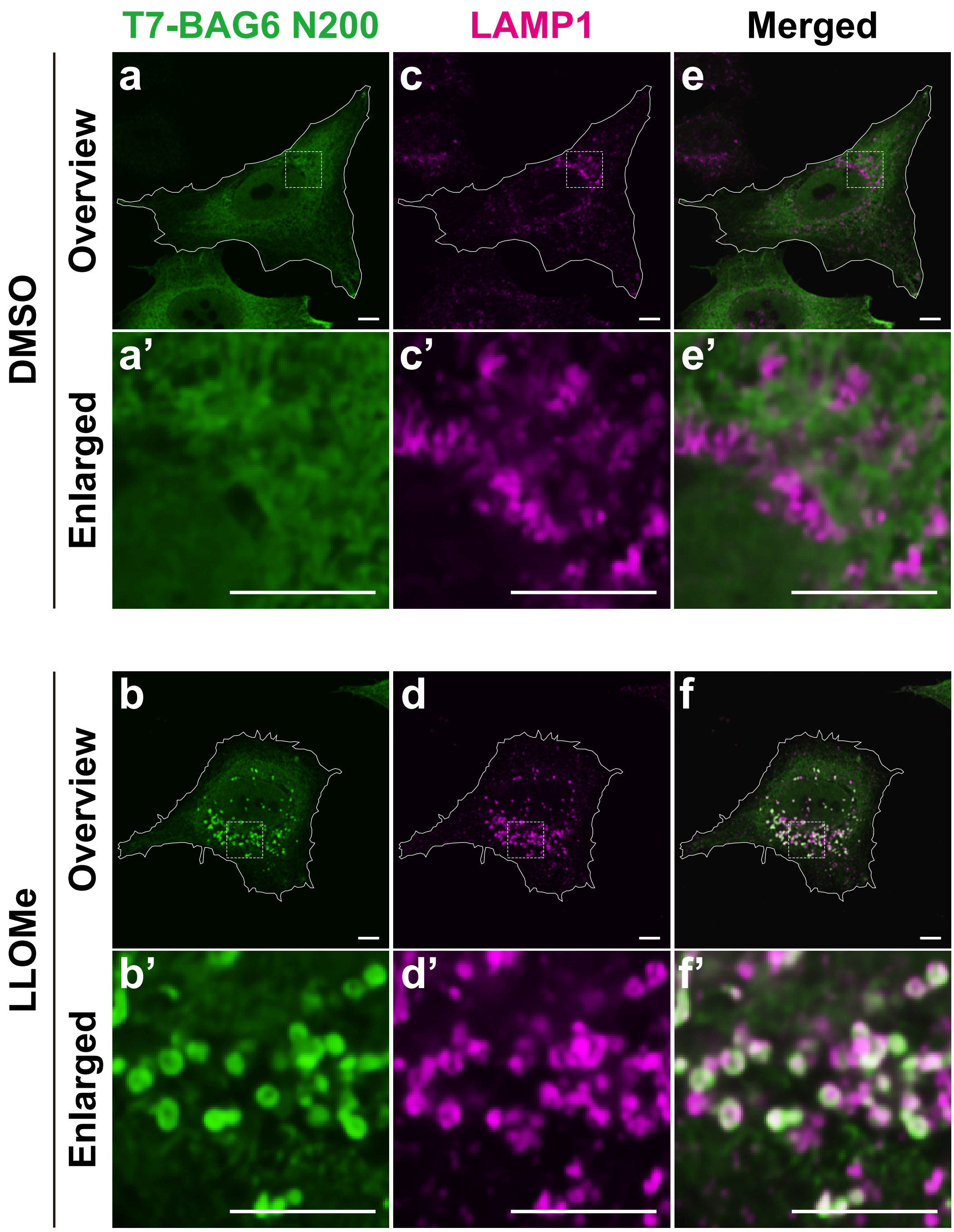

Fig. S3. The substrate-recognition domain of BAG6 (BAG6 N200) is recruited to lysosomes upon LLOMe treatment.

Confocal microscopy observations of T7-BAG6 N200 (T7-tagged N-terminal region of BAG6) in HeLa cells. BAG6 N200 is a fragment required for both substrate recognition and RNF115 association [28,36,59]. Cells were treated with 250 μM LLOMe or its solvent (DMSO) for 1 h and then stained with anti-T7 tag (green) and anti-LAMP1(magenta) antibodies. Typical images from a single experiment are shown. **(a, b)** T7-BAG6 N200 stain. **(c, d)** LAMP1 stain. **(e, f)** Merged images. **(a, c, e)** DMSO-treated cells. **(b, d, f)** LLOMe-treated cells. **(a'–f')** Enlarged views of the areas indicated by rectangles in (a–f), respectively. White lines indicate cell boundaries. Note that all images presented in this figure were acquired in an identical set of experiments and that the exposure times of the respective immunostainings in this figure were the same. T7-tagged BAG6 N200 was found to translocate to the foci close to the lysosome marker LAMP1 following LLOMe-induced lysosomal damage. Some of the BAG6 N200 signals surrounding the LAMP1-positive puncta appeared as vesicle-like structures (b', f'). Scale bar, 5 μm.
